# Supplementary material for: Lagging strand gap suppression connects BRCA-mediated fork protection to nucleosome assembly through PCNA-dependent CAF-1 recycling
Source: Nat Commun. 2022 Sep 9;13:5323. doi: 10.1038/s41467-022-33028-y (PMC9463168; doi:10.1038/s41467-022-33028-y)
Supplement: Supplementary file 1 — Supplementary Information [file 41467_2022_33028_MOESM1_ESM.pdf]

Supplementary Information

Lagging strand gap suppression connects BRCA-mediated fork protection to nucleosome assembly through PCNA-dependent CAF-1 recycling

Thakar et al.

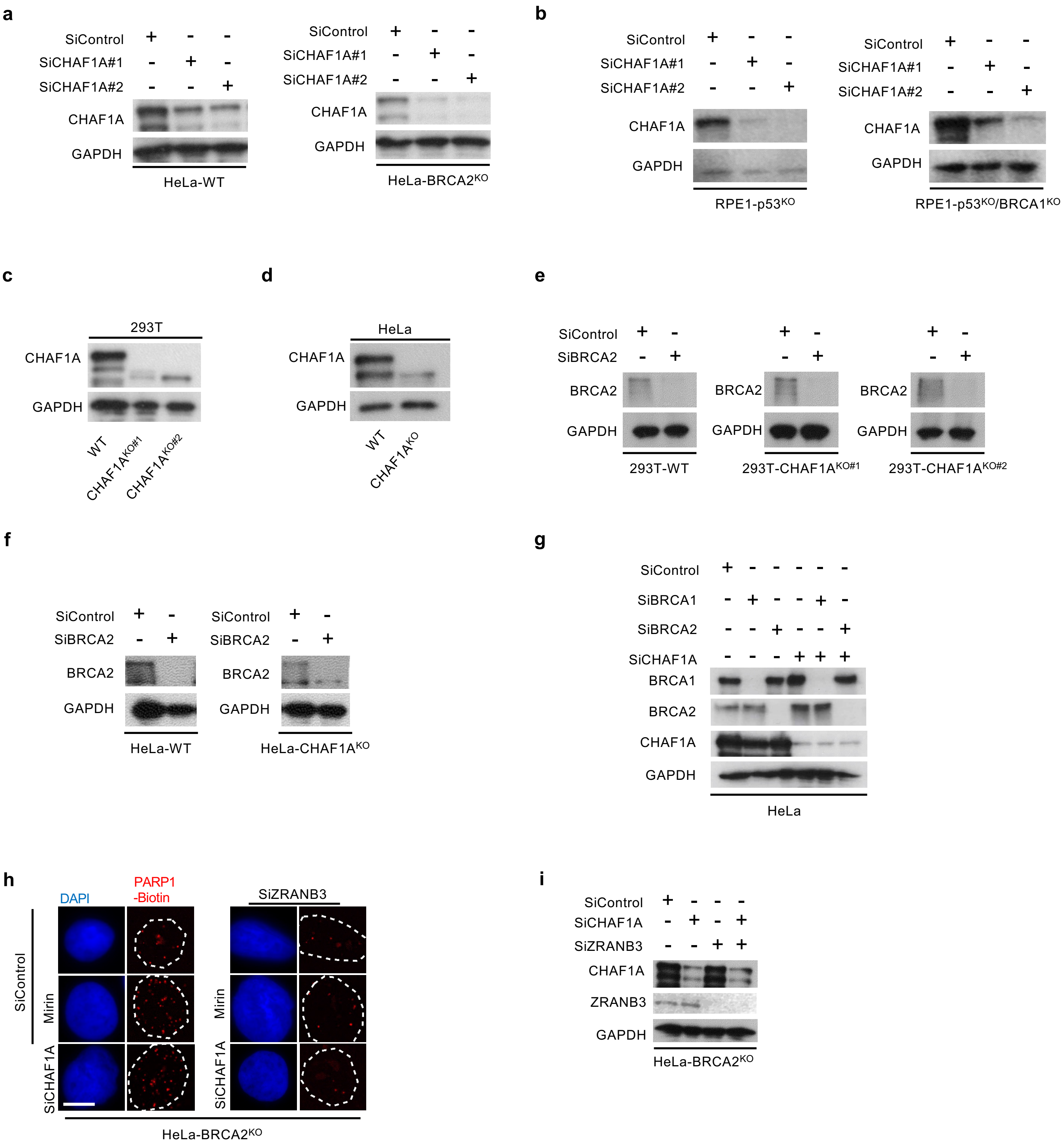
a
SiControl
SiCHAF1A#1
SiCHAF1A#2
CHAF1A
GAPDH
HeLa-WT
HeLa-BRCA2KO
b
RPE1-p53KO
RPE1-p53KO/BRCA1KO
c
293T
WT
CHAF1AKO#1
CHAF1AKO#2
d
HeLa
CHAF1AKO
e
SiBRCA2
BRCA2
293T-WT
293T-CHAF1AKO#1
293T-CHAF1AKO#2
f
HeLa-CHAF1AKO
g
SiBRCA1
SiCHAF1A
BRCA1
HeLa
h
DAPI
PARP1
-Biotin
SiZRANB3
Mirin
i
SiZRANB3
ZRANB3

Supplementary Figure 1. Confirmation of gene knockdowns. a,b. Western blots showing CHAF1A depletion in BRCA2-knockout HeLa cells (**a**) and in BRCA1-knockout RPE1 cells (**b**). **c,d**. Western blots showing CHAF1A knockout in 293T (**c**) and HeLa (**d**) cells. **e,f**. Western blots showing BRCA2 depletion in CHAF1A-knockout 293T (**e**) and HeLa (**f**) cells. **g**. Western blots showing CHAF1A co-depletion with BRCA1 or BRCA2 in HeLa cells. **h**. Representative micrographs of the PARP1 SIRF experiment (scale bar represents 10μm). **i**. Western blots showing CHAF1A co-depletion with ZRANB3 in HeLa-BRCA2^{KO} cells.

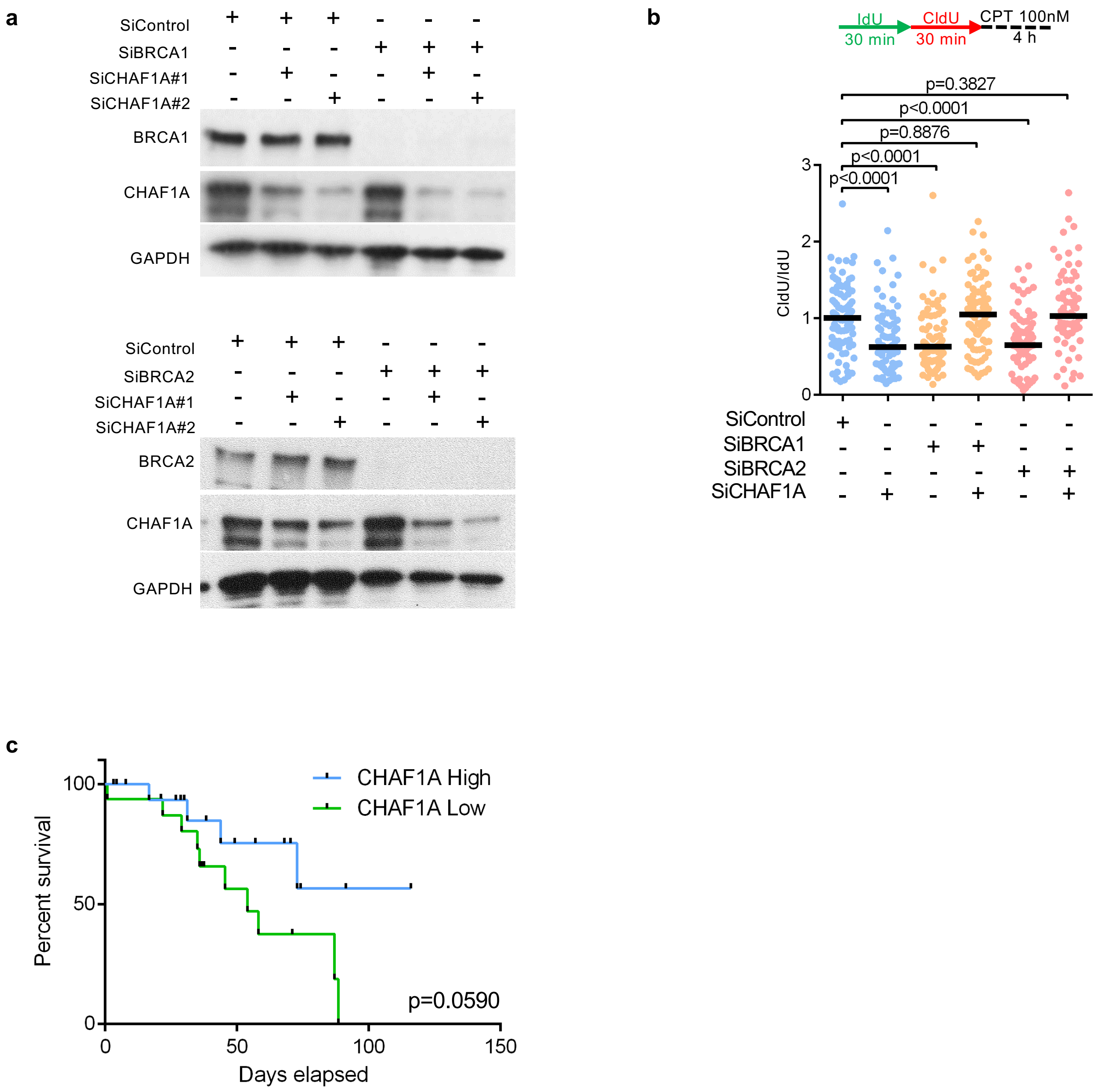

a
SiControl
SiBRCA1
SiCHAF1A#1
SiCHAF1A#2
BRCA1
CHAF1A
GAPDH
SiControl
SiBRCA2
SiCHAF1A#1
SiCHAF1A#2
BRCA2
CHAF1A
GAPDH
b
IdU
30 min
CldU
30 min
CPT 100nM
4 h
p=0.3827
p<0.0001
p=0.8876
p<0.0001
p<0.0001
CldU/IdU
SiControl
SiBRCA1
SiBRCA2
SiCHAF1A
c
CHAF1A High
CHAF1A Low
Percent survival
p=0.0590
Days elapsed

Supplementary Figure 2. Impact of CAF-1 loss in BRCA-deficient cells. a. Western blots showing CHAF1A co-depletion with BRCA1 or BRCA2 in HeLa cells. **b.** DNA fiber combing assay showing that co-depletion of CHAF1A suppresses CPT-induced fork degradation in BRCA1-knockdown and BRCA2-knockdown HeLa cells. The ratio of CldU to IdU tract lengths is presented, with the median values marked on the graph. The p-values (Mann-Whitney test, two-tailed) are listed at the top. A schematic representation of the DNA fiber combing assay conditions is also presented. **c**. Analyses of BRCA2-mutant ovarian TCGA cancer dataset showing that high CHAF1A levels are associated with increased survival, while low CHAF1A levels are associated with reduced survival. Mantel-Cox log ranked t test was used for statistical analyses (n=14, p=0.0590). The difference observed is not significant, likely because of the small number of BRCA2-mutant samples in the dataset.

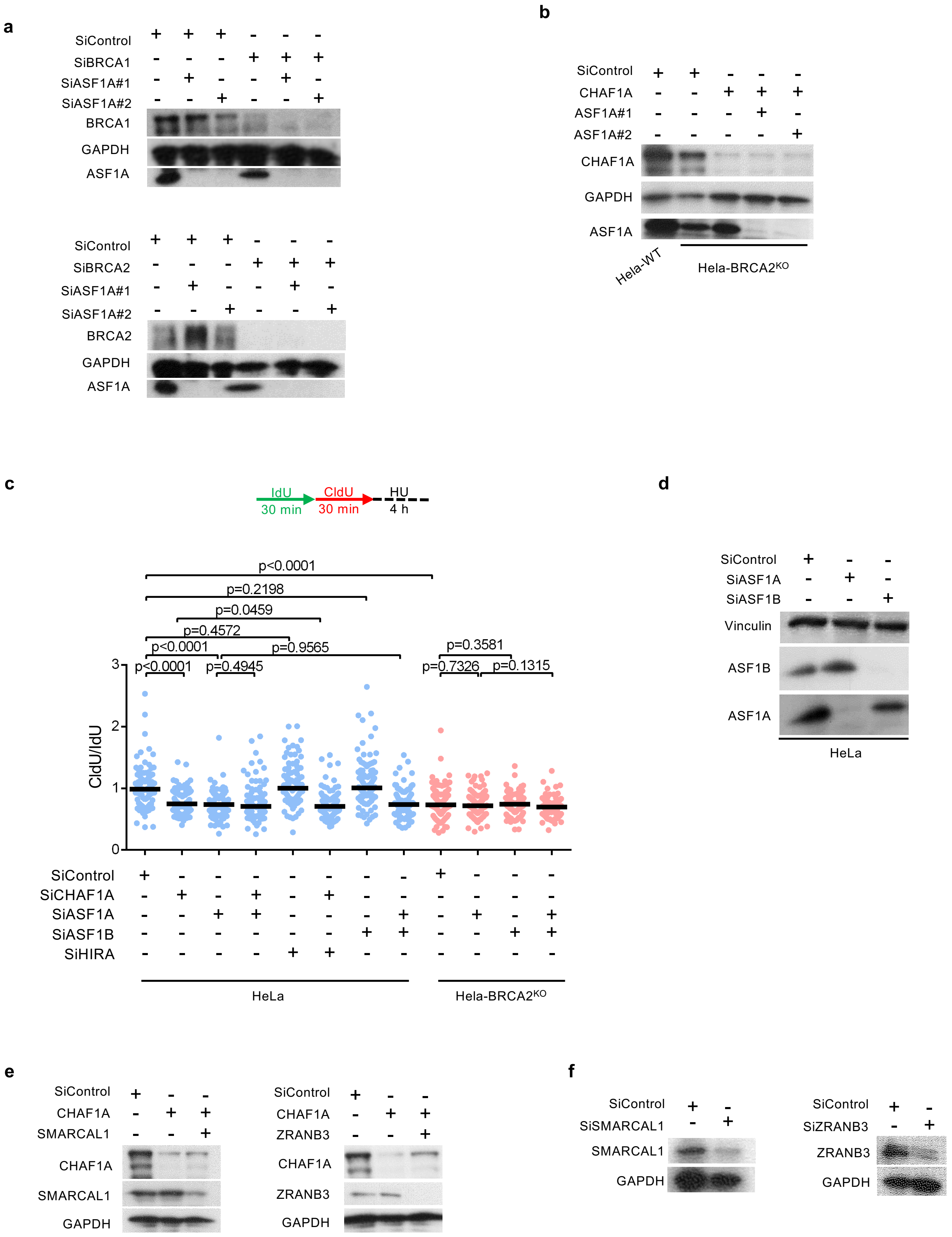

a
SiControl
SiBRCA1
SiASF1A#1
SiASF1A#2
BRCA1
GAPDH
ASF1A
SiControl
SiBRCA2
SiASF1A#1
SiASF1A#2
BRCA2
GAPDH
ASF1A
b
SiControl
CHAF1A
ASF1A#1
ASF1A#2
CHAF1A
GAPDH
ASF1A
Hela-WT
Hela-BRCA2KO
c
IdU
30 min
CldU
30 min
HU
4 h
p<0.0001
p=0.2198
p=0.0459
p=0.4572
p<0.0001
p=0.9565
p<0.0001
p=0.4945
p=0.3581
p=0.7326
p=0.1315
CldU/IdU
SiControl
SiCHAF1A
SiASF1A
SiASF1B
SiHIRA
HeLa
Hela-BRCA2KO
d
SiControl
SiASF1A
SiASF1B
Vinculin
ASF1B
ASF1A
HeLa
e
SiControl
CHAF1A
SMARCAL1
CHAF1A
SMARCAL1
GAPDH
SiControl
CHAF1A
ZRANB3
CHAF1A
ZRANB3
GAPDH
f
SiControl
SiSMARCAL1
SMARCAL1
GAPDH
SiControl
SiZRANB3
ZRANB3
GAPDH

Supplementary Figure 3. Confirmation of gene knockdowns. a. Western blots showing ASF1A co-depletion with BRCA1 or BRCA2 in HeLa cells. **b.** Western blots showing ASF1A co-depletion with CHAF1A in HeLa-BRCA2^{KO} cells. **c.** DNA fiber combing assay showing that ASF1B does not impact HU-induced fork degradation in wildtype or BRCA2-knockout HeLa cells, and that co-depletion of HIRA or of ASF1A does not affect fork degradation induced by CHAF1A knockdown in wildtype HeLa cells. The ratio of CldU to IdU tract lengths is presented, with the median values marked on the graph. The p-values (Mann-Whitney test, two-tailed) are listed at the top. A schematic representation of the DNA fiber combing assay conditions is also presented. **d.** Western blots showing ASF1A and ASF1B knockdowns in HeLa cells. **e.** Western blots showing CHAF1A co-depletion with SMARCAL1 or ZRANB3 in HeLa cells. **f.** Western blots showing ZRANB3 and SMARCAL1 depletions in 293T-CHAF1A^{KO} cells.

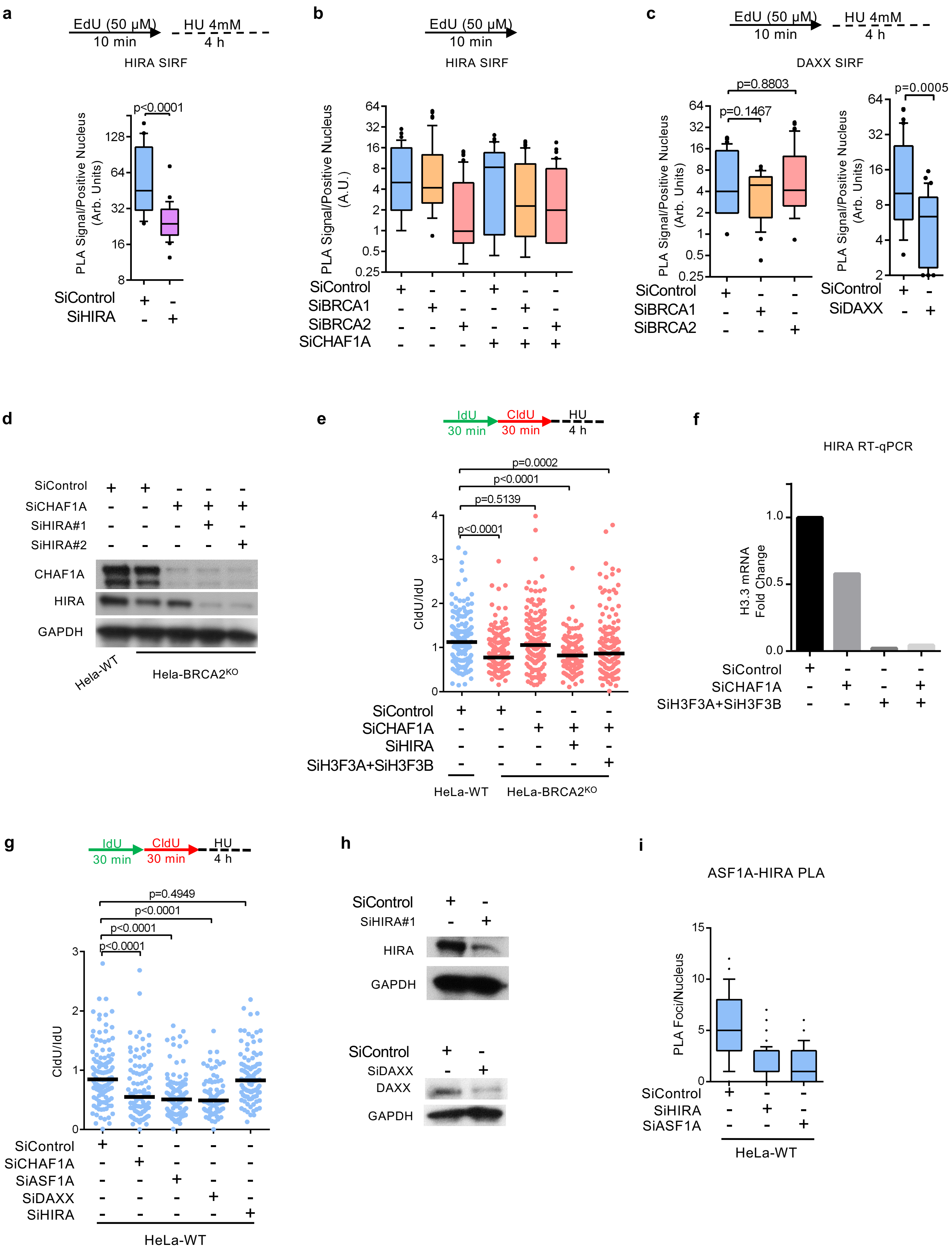
a
EdU (50 μM)
10 min
HU 4mM
4 h
HIRA SIRF
p<0.0001
PLA Signal/Positive Nucleus (Arb. Units)
SiControl
SiHIRA
b
HIRA SIRF
PLA Signal/Positive Nucleus (A.U.)
SiBRCA1
SiBRCA2
SiCHAF1A
c
DAXX SIRF
p=0.8803
p=0.1467
p=0.0005
SiDAXX
d
SiHIRA#1
SiHIRA#2
CHAF1A
HIRA
GAPDH
Hela-WT
Hela-BRCA2KO
e
IdU
30 min
CldU
30 min
HU
4 h
p=0.0002
p<0.0001
p=0.5139
CldU/IdU
SiH3F3A+SiH3F3B
HeLa-WT
HeLa-BRCA2KO
f
HIRA RT-qPCR
H3.3 mRNA Fold Change
g
p=0.4949
SiASF1A
h
DAXX
i
ASF1A-HIRA PLA
PLA Foci/Nucleus

Supplementary Figure 4. Impact of nucleosome deposition by HIRA on BRCA-deficient cells. a. SIRF assay confirm that the HIRA PLA signal is specific, since it is reduced upon HIRA depletion by siRNA. At least 20 positive cells were quantified for each condition. Center line indicates the median, bounds of box indicate the first and third quartile, and whiskers indicate the 10th and 90th percentile. The p-values (Mann-Whitney test, two-tailed) are listed at the top. A schematic representation of the SIRF assay conditions is also presented. **b**. SIRF assay showing the impact of CHAF1A co-depletion on HIRA binding to nascent DNA in BRCA1 or BRCA2-knockdown HeLa cells under normal conditions. At least 35 positive cells were quantified for each condition. Center line indicates the median, bounds of box indicate the first and third quartile, and whiskers indicate the 10th and 90th percentile. A schematic representation of the SIRF assay conditions is also presented. **c**. SIRF assay showing that DAXX binding to nascent DNA is unchanged upon BRCA1 or BRCA2 depletion in HeLa cells. DAXX depletion by siRNA reduces the PLA signal, confirming its specificity. A schematic representation of SIRF assay conditions is also presented. At least 30 positive cells were quantified for each condition. Center line indicates the median, bounds of box indicate the first and third quartile, and whiskers indicate the 10th and 90th percentile. The p-values (Mann-Whitney test, two-tailed) are listed at the top. A schematic representation of the SIRF assay conditions is also presented. **d.** Western blots showing HIRA co-depletion with CHAF1A in HeLa-BRCA2^{KO} cells. **e.** DNA fiber combing assays showing that co-depletion of HIRA or of H3.3-encoding genes H3F3A and H3F3B restores HU-induced nascent strand degradation in CHAF1A-depleted HeLa-BRCA2^{KO} cells. The ratio of CldU to IdU tract lengths is presented, with the median values marked on the graph. The p-values (Mann-Whitney test, two-tailed) are listed at the top. A schematic representation of the DNA fiber combing assay conditions is also presented. **f**. RT-qPCR experiment showing reduction in H3F3A and H3F3B mRNA levels upon siRNA-mediated knockdown. The average of two technical replicates is shown. (No antibody was available to us for verifying the depletion by Western blot.) **g,h.** DNA fiber combing assays

showing the impact of the depletion of histone chaperones HIRA and DAXX on HU-induced nascent strand degradation in HeLa cells. The ratio of CldU to IdU tract lengths is presented, with the median values marked on the graph (**g**). The p-values (Mann-Whitney test, two-tailed) are listed at the top. A schematic representation of the DNA fiber combing assay conditions is also presented. Western blots confirming the knockdowns (**h**) are also presented. **i.** PLA assay in HeLa cells treated with HU (4mM for 3h), demonstrating the specificity of the ASF1A-HIRA PLA signal, since knockdown of HIRA or of ASF1A suppresses this signal. At least 75 cells were quantified for each condition. Center line indicates the median, bounds of box indicate the first and third quartile, and whiskers indicate the 10th and 90th percentile.

a

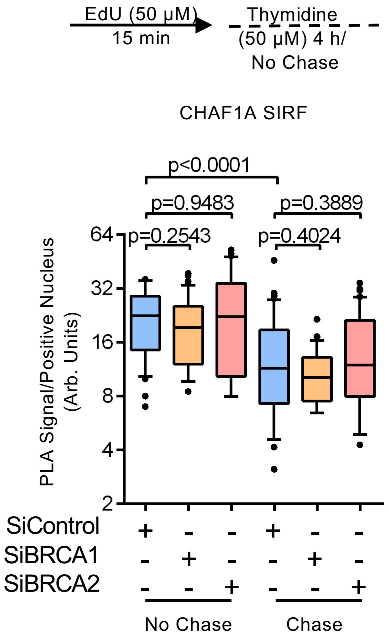
EdU (50 μM)
15 min
Thymidine
(50 μM) 4 h/
No Chase
CHAF1A SIRF
p<0.0001
p=0.9483
p=0.3889
p=0.2543
p=0.4024
PLA Signal/Positive Nucleus
(Arb. Units)
64
32
16
8
4
2
SiControl
SiBRCA1
SiBRCA2
No Chase
Chase

b

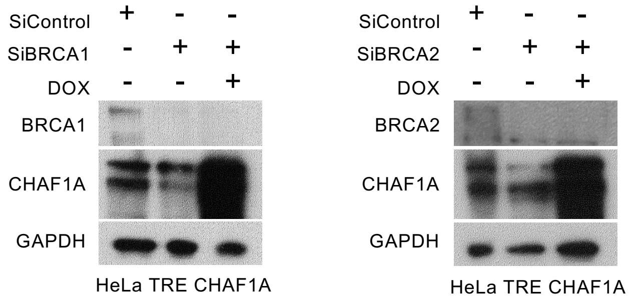
SiControl
SiBRCA1
DOX
BRCA1
CHAF1A
GAPDH
HeLa TRE CHAF1A
SiControl
SiBRCA2
DOX
BRCA2
CHAF1A
GAPDH
HeLa TRE CHAF1A

Supplementary Figure 5. CHAF1A recycling in BRCA-deficient cells. a. SIRF assay showing that, in the absence of replication stress, CHAF1A recycling is not impaired in BRCA1 or BRCA2-depleted HeLa cells. Cells were labeled with EdU, washed, and chased for 4h in fresh media containing 50μM thymidine. At least 25 positive cells were quantified for each condition. Center line indicates the median, bounds of box indicate the first and third quartile, and whiskers indicate the 10th and 90th percentile. The p-values (Mann-Whitney test, two-tailed) are listed at the top. A schematic representation of the SIRF assay conditions is also presented. **b.** Western blots showing doxycycline-induced CHAF1A overexpression, and BRCA1 or BRCA2 knockdown in these cells.

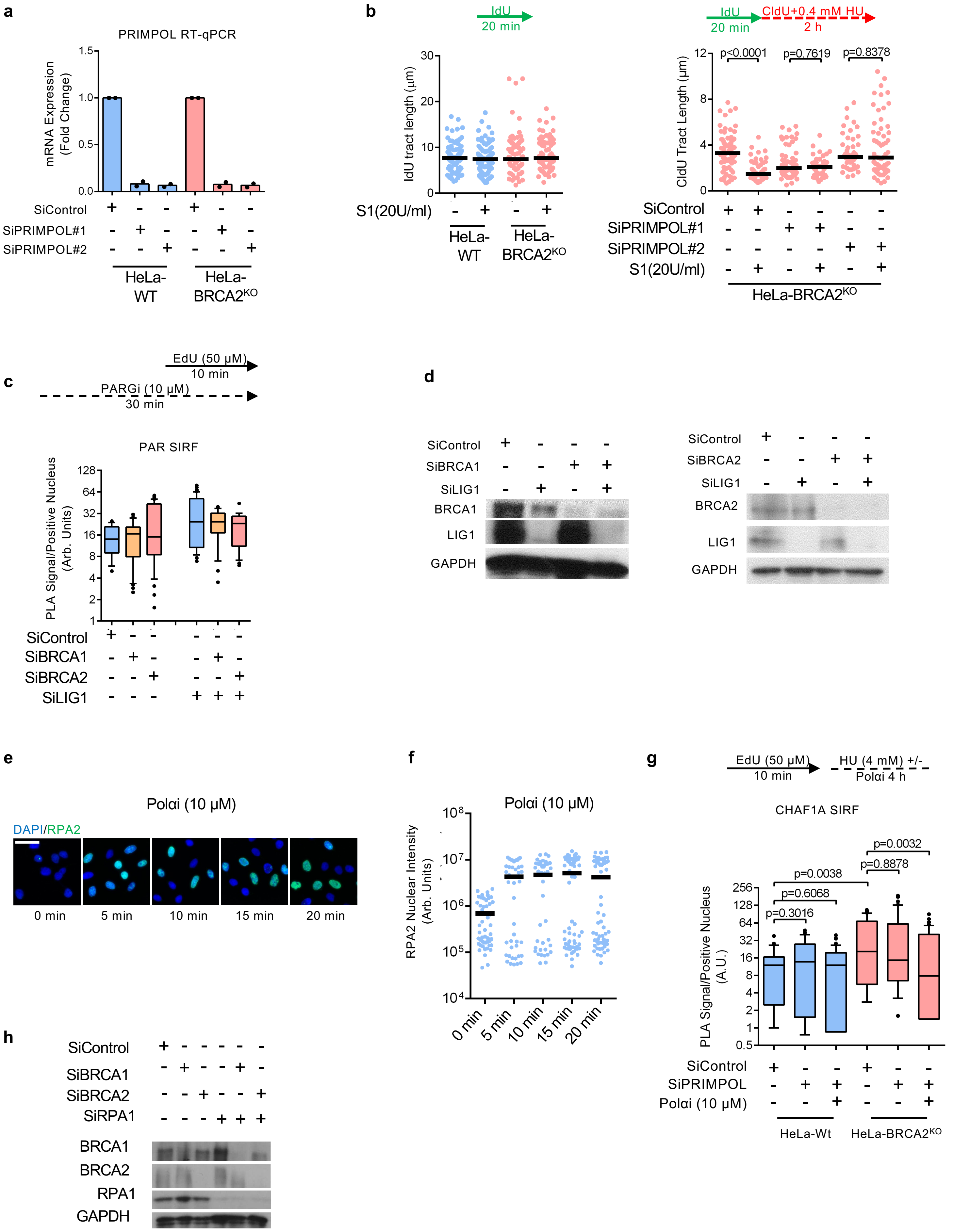
a
PRIMPOL RT-qPCR
mRNA Expression (Fold Change)
1.5
1.0
0.5
0.0
SiControl + - - + - -
SiPRIMPOL#1 - + - - + -
SiPRIMPOL#2 - - + - - +
HeLa-WT
HeLa-BRCA2KO
b
IdU 20 min
IdU tract length (µm)
30
20
10
0
S1(20U/ml) - + - +
HeLa-WT
HeLa-BRCA2KO
IdU 20 min
CldU+0.4 mM HU 2 h
CldU Tract Length (µm)
12
8
4
0
p<0.0001
p=0.7619
p=0.8378
SiControl + + - - - -
SiPRIMPOL#1 - - + + - -
SiPRIMPOL#2 - - - - + +
S1(20U/ml) - + - + - +
HeLa-BRCA2KO
c
EdU (50 µM) 10 min
PARGi (10 µM) 30 min
PAR SIRF
PLA Signal/Positive Nucleus (Arb. Units)
128
64
32
16
8
4
2
1
SiControl + - - - - -
SiBRCA1 - + - - + -
SiBRCA2 - - + - - +
SiLIG1 - - - + + +
d
SiControl + - - -
SiBRCA1 - - + +
SiLIG1 - + - +
BRCA1
LIG1
GAPDH
SiControl + - - -
SiBRCA2 - - + +
SiLIG1 - + - +
BRCA2
LIG1
GAPDH
e
Polαi (10 µM)
DAPI/RPA2
0 min
5 min
10 min
15 min
20 min
f
Polαi (10 µM)
RPA2 Nuclear Intensity (Arb. Units)
10^8
10^7
10^6
10^5
10^4
0 min
5 min
10 min
15 min
20 min
g
EdU (50 µM) 10 min
HU (4 mM) +/- Polαi 4 h
CHAF1A SIRF
PLA Signal/Positive Nucleus (A.U.)
256
128
64
32
16
8
4
2
1
0.5
p=0.0032
p=0.8878
p=0.0038
p=0.6068
p=0.3016
SiControl + - - + - -
SiPRIMPOL - + + - + +
Polαi (10 µM) - - + - - +
HeLa-Wt
HeLa-BRCA2KO
h
SiControl + - - - - -
SiBRCA1 - + - - + -
SiBRCA2 - - + - - +
SiRPA1 - - - + + +
BRCA1
BRCA2
RPA1
GAPDH

Supplementary Figure 6. Confirmation of gene knockdowns. a. RT-qPCR experiment showing reduction in PRIMPOL mRNA levels upon siRNA-mediated knockdown. The average of two biological replicates is shown. (No antibody was available to us for verifying the depletion by Western blot.) **b.** S1 nuclease fiber spreading assays showing that fork shortening upon HU-induced replication stress is completely rescued by PRIMPOL depletion in HeLa-BRCA2^{KO} cells. The p-values (Mann-Whitney test, two-tailed) are listed at the top. A schematic representation of the DNA fiber spreading assay conditions is also presented. **c.** SIRF assay showing that, under normal growth conditions (in the absence of replication stress), LIG1 knockdown induces PAR chain formation similarly in wildtype and BRCA1 or BRCA2-depleted HeLa cells. At least 25 positive cells were quantified for each condition. Center line indicates the median, bounds of box indicate the first and third quartile, and whiskers indicate the 10th and 90th percentile. A schematic representation of the SIRF assay conditions is also presented. **d.** Western blots showing LIG1 co-depletion with BRCA1 or BRCA2 in HeLa cells. **e,f**. Immunofluorescence experiment showing the impact of Polα inhibition by treatment with 10μM ST1926 on RPA2 chromatin foci in HeLa cells. Representative micrographs (scale bar represents 50μm) (**e**) and quantifications (**f**) are shown. At least 40 cells were quantified for each condition. The mean values are represented on the graph. **g.** SIRF assay showing that CHAF1A retention on nascent DNA in BRCA-deficient cells is not affected by PRIMPOL depletion. At least 40 positive cells were quantified for each condition. Center line indicates the median, bounds of box indicate the first and third quartile, and whiskers indicate the 10th and 90th percentile. The p-values (Mann-Whitney test, two-tailed) are listed at the top. A schematic representation of the SIRF assay conditions is also presented. **h.** Western blots showing RPA1 co-depletion with BRCA1 or BRCA2 in HeLa cells.

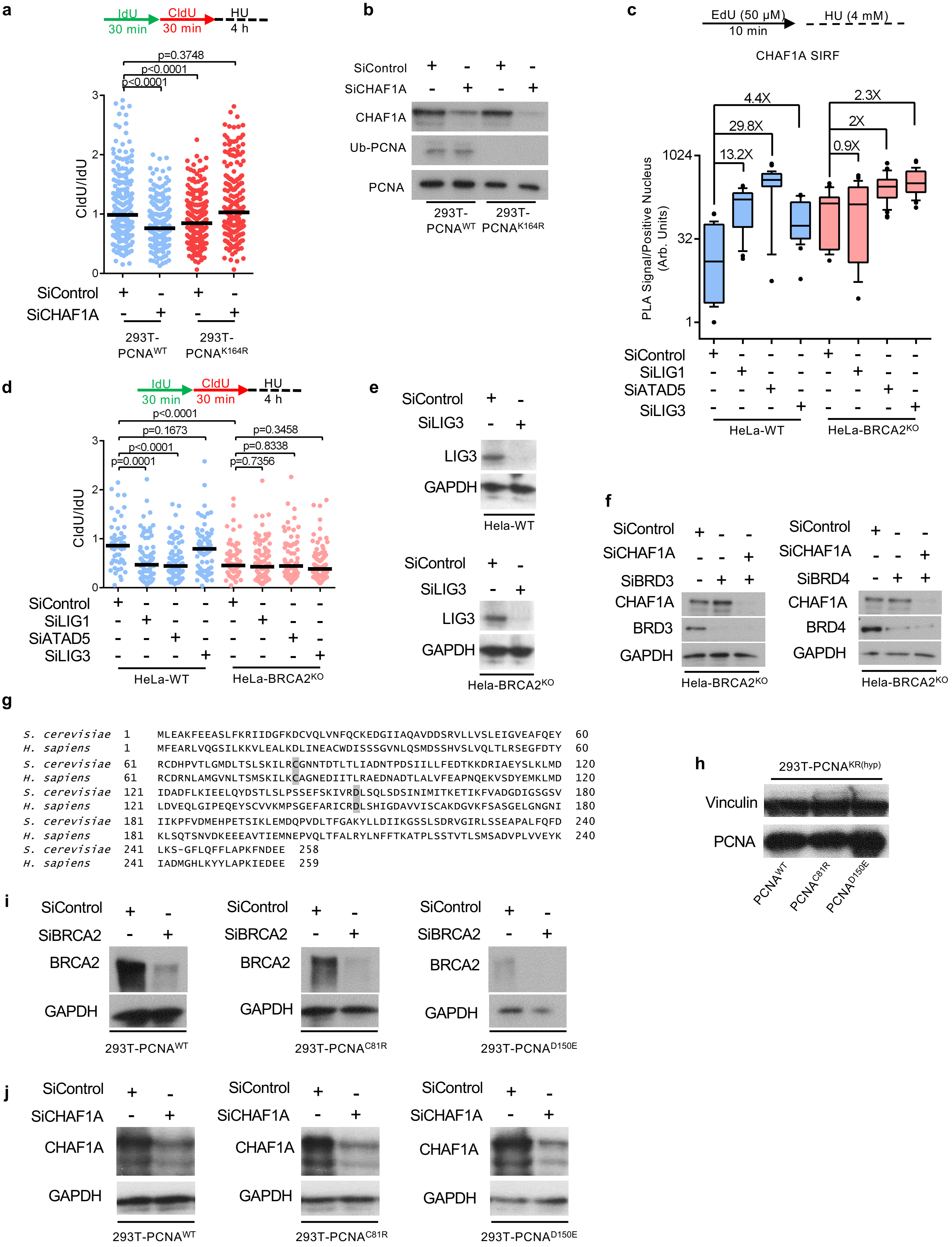
a
IdU 30 min
CldU 30 min
HU 4 h
p=0.3748
p<0.0001
p<0.0001
CldU/IdU
SiControl + - + -
SiCHAF1A - + - +
293T-PCNA^WT
293T-PCNA^K164R
b
SiControl + - + -
SiCHAF1A - + - +
CHAF1A
Ub-PCNA
PCNA
293T-PCNA^WT
293T-PCNA^K164R
c
EdU (50 μM) 10 min
HU (4 mM)
CHAF1A SIRF
PLA Signal/Positive Nucleus (Arb. Units)
4.4X
29.8X
13.2X
2.3X
2X
0.9X
SiControl
SiLIG1
SiATAD5
SiLIG3
HeLa-WT
HeLa-BRCA2^KO
d
IdU 30 min
CldU 30 min
HU 4 h
p<0.0001
p=0.1673
p<0.0001
p=0.0001
p=0.3458
p=0.8338
p=0.7356
CldU/IdU
SiControl
SiLIG1
SiATAD5
SiLIG3
HeLa-WT
HeLa-BRCA2^KO
e
SiControl + -
SiLIG3 - +
LIG3
GAPDH
Hela-WT
SiControl + -
SiLIG3 - +
LIG3
GAPDH
Hela-BRCA2^KO
f
SiControl + - -
SiCHAF1A - - +
SiBRD3 - + +
CHAF1A
BRD3
GAPDH
Hela-BRCA2^KO
SiControl + - -
SiCHAF1A - - +
SiBRD4 - + +
CHAF1A
BRD4
GAPDH
Hela-BRCA2^KO
g
S. cerevisiae 1 MLEAKFEEASLFKRIIDGFKDCVQLVNFQCKEDGIIAQAVDDSRVLLVSLEIGVEAFQEY 60
H. sapiens 1 MFEARLVQGSILKKVLEALKDLINEACWDISSSGVNLQSMDSSHVSLVQLTLRSEGFDTY 60
S. cerevisiae 61 RCDHPVTLGMDLTSLSKILRCGNNTDTLTLIADNTPDSIILLFEDTKKDRIAEYSLKLMD 120
H. sapiens 61 RCDRNLAMGVNLTSMSKILKCAGNEDIITLRAEDNADTLALVFEAPNQEKVSDYEMKLMD 120
S. cerevisiae 121 IDADFLKIEELQYDSTLSLPSSEFSKIVRDLSQLSDSINIMITKETIKFVADGDIGSGSV 180
H. sapiens 121 LDVEQLGIPEQEYSCVVKMPSGEFARICRDLSHIGDAVVISCAKDGVKFSASGELGNGNI 180
S. cerevisiae 181 IIKPFVDMEHPETSIKLEMDQPVDLTFGAKYLLDIIKGSSLSDRVGIRLSSEAPALFQFD 240
H. sapiens 181 KLSQTSNVDKEEEAVTIEMNEPVQLTFALRYLNFFTKATPLSSTVTLSMSADVPLVVEYK 240
S. cerevisiae 241 LKS-GFLQFFLAPKFNDEE 258
H. sapiens 241 IADMGHLKYYLAPKIEDEE 259
h
293T-PCNA^KR(hyp)
Vinculin
PCNA
PCNA^WT
PCNA^C81R
PCNA^D150E
i
SiControl + -
SiBRCA2 - +
BRCA2
GAPDH
293T-PCNA^WT
293T-PCNA^C81R
293T-PCNA^D150E
j
SiControl + -
SiCHAF1A - +
CHAF1A
GAPDH
293T-PCNA^WT
293T-PCNA^C81R
293T-PCNA^D150E

Supplementary Figure 7. Confirmation of gene knockdowns. a. DNA fiber combing assays showing that CHAF1A depletion results in HU-induced fork degradation in wildtype cells, but suppresses fork degradation in 293T-PCNA^{K164R} cells. The ratio of CldU to IdU tract lengths is presented, with the median values marked on the graph. The p-values (Mann-Whitney test, two-tailed) are listed at the top. A schematic representation of the DNA fiber combing assay conditions is also presented. **b**. Western blots showing CHAF1A depletion in 293T cells. **c**. SIRF assay showing CHAF1A retention on nascent DNA in wildtype and BRCA2-knockout HeLa cells upon depletion of LIG1, LIG3 or ATAD5. At least 25 positive cells were quantified for each condition. The fold differences are listed. Center line indicates the median, bounds of box indicate the first and third quartile, and whiskers indicate the 10th and 90th percentile. A schematic representation of the SIRF assay conditions is also presented. **d**. DNA fiber combing assays showing the impact of LIG1, LIG3, and ATAD5 depletion on HU-induced fork degradation in wildtype and BRCA2-knockout HeLa cells. The ratio of CldU to IdU tract lengths is presented, with the median values marked on the graph. The p-values (Mann-Whitney test, two-tailed) are listed at the top. Schematic representations of the DNA fiber combing assay conditions are also presented. At least 50 tracts were quantified for each condition. **e.** Western blots showing LIG3 depletion in wildtype and BRCA2-knockout HeLa cells. **f.** Western blots showing CHAF1A co-depletion with BRCA3 or BRD4 in HeLa-BRCA2^{KO} cells. **g.** Alignment of yeast and human PCNA, indicating the PCNA mutations performed. **h**. Western blots showing the expression of PCNA variants in PCNA-hypomorph 293T cells. **i.** Western blots showing BRCA2 depletion in 293T cells expressing PCNA variants. **j.** Western blots showing CHAF1A depletion in 293T cells expressing PCNA variants.

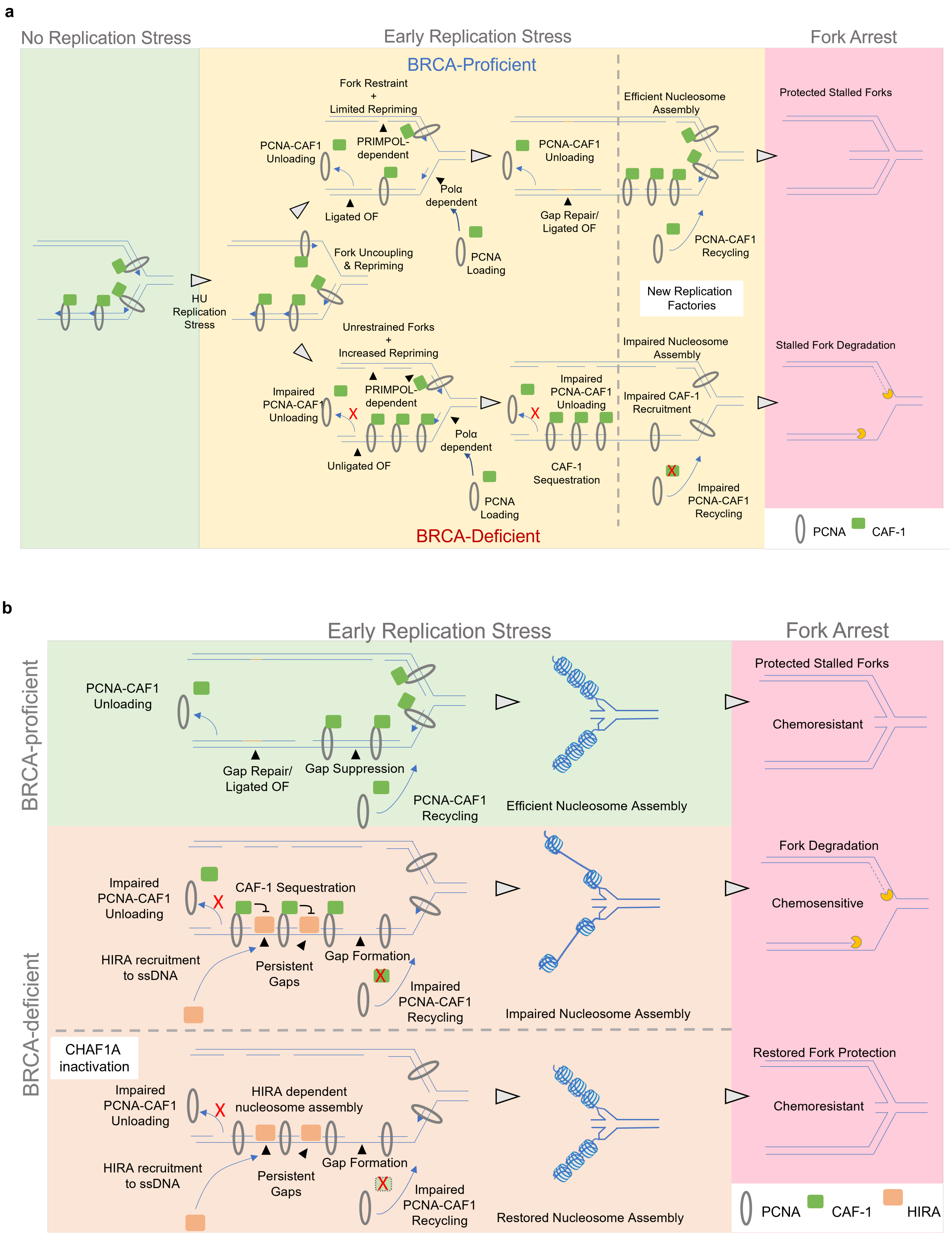
a
No Replication Stress
Early Replication Stress
Fork Arrest
BRCA-Proficient
Fork Restraint
+
Limited Repriming
PCNA-CAF1 Unloading
PRIMPOL-dependent
Ligated OF
Polα dependent
PCNA Loading
PCNA-CAF1 Unloading
Gap Repair/ Ligated OF
Efficient Nucleosome Assembly
PCNA-CAF1 Recycling
New Replication Factories
Protected Stalled Forks
HU Replication Stress
Fork Uncoupling & Repriming
Unrestrained Forks
+
Increased Repriming
Impaired PCNA-CAF1 Unloading
PRIMPOL-dependent
Unligated OF
Polα dependent
PCNA Loading
Impaired PCNA-CAF1 Unloading
CAF-1 Sequestration
Impaired Nucleosome Assembly
Impaired CAF-1 Recruitment
Impaired PCNA-CAF1 Recycling
Stalled Fork Degradation
BRCA-Deficient
PCNA
CAF-1
b
Early Replication Stress
Fork Arrest
BRCA-proficient
PCNA-CAF1 Unloading
Gap Repair/ Ligated OF
Gap Suppression
PCNA-CAF1 Recycling
Efficient Nucleosome Assembly
Protected Stalled Forks
Chemoresistant
BRCA-deficient
Impaired PCNA-CAF1 Unloading
CAF-1 Sequestration
HIRA recruitment to ssDNA
Persistent Gaps
Gap Formation
Impaired PCNA-CAF1 Recycling
Impaired Nucleosome Assembly
Fork Degradation
Chemosensitive
CHAF1A inactivation
Impaired PCNA-CAF1 Unloading
HIRA dependent nucleosome assembly
HIRA recruitment to ssDNA
Persistent Gaps
Gap Formation
Impaired PCNA-CAF1 Recycling
Restored Nucleosome Assembly
Restored Fork Protection
Chemoresistant
PCNA
CAF-1
HIRA

Supplementary Figure 8. Schematic representations of the proposed models. a. Model for CAF-1 recycling defects as the underling factor responsible for fork degradation in BRCA-deficient cells. In BRCA-proficient cells, stressed replication forks are slowed, minimizing replication-associated gap formation. BRCA-deficient cells show impaired fork restraint during replication stress, instead undergoing repriming. On the leading strand, forks are reprimed by PRIMPOL leaving behind leading strand gaps. On the lagging strand, forks reprime through Polα-mediated initiation of the subsequent OF, leaving behind lagging strand gaps. Both leading and lagging strand gaps can also be efficiently repaired by BRCA-mediated PRR. On the lagging strand, the BRCA pathway mediates gap suppression and ensures timely OF ligation, allowing unloading of PCNA-CAF-1 complexes and subsequent CAF-1 recycling to ongoing forks. This ensures proper nucleosome assembly, protecting forks from nucleolytic degradation if they arrest and reverse at a later time. In BRCA-deficient cells, gaps on the lagging strand accumulate and retain PCNA-CAF-1 complexes since OF ligation cannot take place. This reduces the availability of CAF-1 at ongoing replication forks, resulting in nucleosome deposition defects. In turn, this predisposes forks to nucleolytic degradation upon their reversal. **b.** Model for restoration of fork stability and chemoresistance upon CAF-1 loss in BRCA-deficient cells. In wildtype cells, efficient gap repair and subsequent OF ligation allows effective CAF-1 recycling and proper nucleosome assembly. In BRCA-deficient cells, ssDNA gaps accumulate and are coated by RPA, which promotes recruitment of HIRA. However, the presence of CAF-1, sequestered in PCNA complexes at lagging strand gaps, inhibits HIRA activity, possibly by competing for ASF1. Loss of CAF-1 releases HIRA activity, resulting in efficient nucleosome assembly and subsequent fork protection.
