# Supplementary material for: Lagging strand gap suppression connects BRCA-mediated fork protection to nucleosome assembly through PCNA-dependent CAF-1 recycling
Source: Nat Commun. 2022 Sep 9;13:5323. doi: 10.1038/s41467-022-33028-y (PMC9463168; doi:10.1038/s41467-022-33028-y)
Supplement: Supplementary file 3 — Reporting Summary [file 41467_2022_33028_MOESM3_ESM.pdf]

nature portfolio

Corresponding author(s): George-Lucian Moldovan

Last updated by author(s): Aug 16, 2022

Reporting Summary

Nature Portfolio wishes to improve the reproducibility of the work that we publish. This form provides structure for consistency and transparency in reporting. For further information on Nature Portfolio policies, see our Editorial Policies and the Editorial Policy Checklist.

Statistics

For all statistical analyses, confirm that the following items are present in the figure legend, table legend, main text, or Methods section.

n/a	Confirmed	
☐	☒	The exact sample size (*n*) for each experimental group/condition, given as a discrete number and unit of measurement
☐	☒	A statement on whether measurements were taken from distinct samples or whether the same sample was measured repeatedly
☐	☒	The statistical test(s) used AND whether they are one- or two-sided<br>*Only common tests should be described solely by name; describe more complex techniques in the Methods section.*
☒	☐	A description of all covariates tested
☒	☐	A description of any assumptions or corrections, such as tests of normality and adjustment for multiple comparisons
☐	☒	A full description of the statistical parameters including central tendency (e.g. means) or other basic estimates (e.g. regression coefficient) AND variation (e.g. standard deviation) or associated estimates of uncertainty (e.g. confidence intervals)
☐	☒	For null hypothesis testing, the test statistic (e.g. *F*, *t*, *r*) with confidence intervals, effect sizes, degrees of freedom and *P* value noted<br>*Give P values as exact values whenever suitable.*
☒	☐	For Bayesian analysis, information on the choice of priors and Markov chain Monte Carlo settings
☒	☐	For hierarchical and complex designs, identification of the appropriate level for tests and full reporting of outcomes
☒	☐	Estimates of effect sizes (e.g. Cohen's *d*, Pearson's *r*), indicating how they were calculated

Our web collection on statistics for biologists contains articles on many of the points above.

Software and code

Policy information about availability of computer code

Data collection	1) DNA Fiber Combing, S1 Nuclease Assay with DNA Fiber Spreading: LASX 3.5.7.23225<br>2) Immunofluorescence, In-Situ Analysis of Protein Interactions at DNA Replication Forks (SIRF): SoftWorx 6.5.2<br>3) Comet Assay: NIS Elements V1.10.00
Data analysis	1) DNA Fiber Combing, S1 Nuclease Assay with DNA Fiber Spreading: LASX 3.5.7.23225<br>2) Immunofluorescence: Fiji (ImageJ2)<br>3) In-Situ Analysis of Protein Interactions at DNA Replication Forks (SIRF): Fiji (ImageJ2)<br>4) Comet Assay: CometScore 2.0<br>5) Statistics and Data Figure Panel: Graphpad Prism 6 and Microsoft Excel v2205

For manuscripts utilizing custom algorithms or software that are central to the research but not yet described in published literature, software must be made available to editors and reviewers. We strongly encourage code deposition in a community repository (e.g. GitHub). See the Nature Portfolio guidelines for submitting code & software for further information.

Data

Policy information about availability of data

All manuscripts must include a data availability statement. This statement should provide the following information, where applicable:

- Accession codes, unique identifiers, or web links for publicly available datasets
- A description of any restrictions on data availability
- For clinical datasets or third party data, please ensure that the statement adheres to our policy

TCGA datasets were obtained from https://www.cbioportal.org. The datasets generated during and/or analysed during the current study are available from the corresponding author on reasonable request. Source data are provided with this paper.

Field-specific reporting

Please select the one below that is the best fit for your research. If you are not sure, read the appropriate sections before making your selection.

☒ Life sciences ☐ Behavioural & social sciences ☐ Ecological, evolutionary & environmental sciences

For a reference copy of the document with all sections, see nature.com/documents/nr-reporting-summary-flat.pdf

Life sciences study design

All studies must disclose on these points even when the disclosure is negative.

Sample size	No statistical method was used to predetermine sample size. Sample size was determined based on previous experiments and relevant literature in the field (eg Taglialatela et al, PMID: 29053959; Lemacon et al, PMID: 29038425; Tirman et al, PMID: 34624216). Statistical significance was obtained with this sample size.
Data exclusions	No data was excluded from the analysis.
Replication	All experiments were performed at least twice. All results were reproducible.
Randomization	Samples were randomly allocated into experimental groups. Samples were processed and analyzed in random order.
Blinding	For imaging-based assays, samples were tested at least once under blinding conditions, and results were reproduced. Blinding was not performed for western blot experiments, since those experiments were simply aimed at validating antibodies and thus blinding was deemed irrelevant.

Reporting for specific materials, systems and methods

We require information from authors about some types of materials, experimental systems and methods used in many studies. Here, indicate whether each material, system or method listed is relevant to your study. If you are not sure if a list item applies to your research, read the appropriate section before selecting a response.

Materials & experimental systems

n/a	Involved in the study	
☐	☒	Antibodies
☐	☒	Eukaryotic cell lines
☒	☐	Palaeontology and archaeology
☒	☐	Animals and other organisms
☒	☐	Human research participants
☒	☐	Clinical data
☒	☐	Dual use research of concern

Methods

n/a	Involved in the study	
☒	☐	ChIP-seq
☒	☐	Flow cytometry
☒	☐	MRI-based neuroimaging

Antibodies

Antibodies used	Antibodies used for Western blot were: CHAF1A (Cell Signaling Technology 5480); ASF1 (Santa Cruz Biotechnology sc-53171); BRCA1 (Santa Cruz Biotechnology sc-6954); BRCA2 (Calbiochem OP95); ZRANB3 (Invitrogen PA5-65143); SMARCAL1 (Invitrogen PA5-54181); HIRA (Abcam 129169); DAXX (Invitrogen PA5-79137); RPA1 (Cell Signaling Technology 2198); LIG1 (Bethyl A301-136A); PRIMPOL (Invitrogen MA5-32899); MRE11 (GeneTex GTX70212); PCNA (Cell Signaling Technology 2586); ubiquitinated PCNA (Cell Signaling Technology 13439); BRD3 (Bethyl A302-368A); BRD4 (Bethyl A700-005); GAPDH (Santa Cruz Biotechnology sc-47724); LIG3 (Santa Cruz Biotechnology sc-135883); ASF1B (Cell Signaling Technology 2769). All antibodies were used at a dilution of 1:500.<br><br>Antibodies used for Immunofluorescence were: Primary antibodies: RAD51 (Abcam ab133534); gH2AX (Millipore 05-636); RPA2

(Abcam ab2175). Secondary antibodies: AlexaFluor 488 or AlexaFluor 568 (Invitrogen A11001, A11008, A11031, and A11036).

Primary Antibodies used for SIRF assays were: Biotin (mouse: Jackson ImmunoResearch 200-002-211; rabbit: Bethyl Laboratories A150-109A); PARP1 (Cell Signaling Technology 9542); CHAF1A (Cell Signaling Technology 5480); HIRA (Abcam 129169); DAXX (Invitrogen PA5-79137); PAR (R&D systems 4335-MC-100); PCNA (Cell Signaling Technology 13110); Histone H3 (Cell Signaling Technology 4499). Antibodies used for PLA were: ASF1A (Santa Cruz Biotechnology sc-53171) and HIRA (Novus NBP3-04893).

Antibodies used for DNA fiber combing were: CldU (Abcam 6236) and IdU (BD 347580) and secondary Cy3 and Cy5 (Abcam 6946 and Abcam 6565).

Validation	Antibodies were validated by western blots or imaging-based assays using siRNA-mediated knockdown or CRISPR-mediated knockout. The data is presented in the manuscript (Supplementary Fig. S1a-g, S1i, S2a, S3a,b,d,e,f, S4a,c,d,h,i, S5b, S6d,h, S7b,e,f,i,j).

Eukaryotic cell lines

Policy information about cell lines

Cell line source(s)	HeLa and 293T cells were obtained from ATCC. For CHAF1A gene knockout, the commercially available CHAF1A CRISPR/Cas9 KO plasmid was used (Santa Cruz Biotechnology sc-402472). The BRCA2-knockout HeLa cells were created in our laboratory and were previously described (Clements et al., 2018). RPE1 and RPE1-p53KO-BRCA1KO were obtained from Dr. Alan D'Andrea (Dana-Farber Cancer Institute, Boston, MA) (Lim et al., 2018). 293T cells with hypomorph PCNA expression were created in our laboratory and previously described (Thakar et al., 2020). For exogenous PCNA expression, pLV[Exp]-Puro-CMV lentiviral constructs encoding wildtype or the indicated variants were obtained from Cyagen. For doxycycline-induced CHAF1A overexpression, the pLV[Exp]-Puro-TRE>hCHAF1A lentiviral construct (Cyagen) was used. Infected cells were selected by puromycin.
Authentication	Authentication was performed regularly based on morphology and gene/protein expression (in case of genetic alterations)
Mycoplasma contamination	Cell lines tested negative for mycoplasma.
Commonly misidentified lines (See ICLAC register)	None of the cell lines used are listed on the ICLAC register version 10
